# Is the Intradural Disc Really Intradural?

**DOI:** 10.7759/cureus.76128

**Published:** 2024-12-21

**Authors:** Ahmed El-Sherif, Mostafa A Mostafa

**Affiliations:** 1 Neurosurgery, Al-Azhar University, Giza, EGY

**Keywords:** cauda equina syndrome (ces), chronic inflammation, intradural disc herniation, lumbar spine pathology, surgical intervention

## Abstract

Intradural disc herniation (IDDH) is a rare condition, accounting for less than 0.5% of herniated disc cases, primarily affecting the lumbar region and often presenting with severe nerve compression or cauda equina syndrome. This paper presents the case of a 60-year-old female with a history of hypertension, dyslipidemia, stroke, and hypothyroidism, who arrived with severe lower back pain, lower limb weakness, and urinary retention. MRI indicated a posterior dural-based mass with significant cauda equina compression and lumbar instability at L3-L4. During surgery, an IDDH was suspected due to adhesions, initially resembling a meningioma, and was confirmed histopathologically. Following excision and spinal fixation, the patient experienced substantial improvement in motor function and pain levels. This case underscores the need to consider IDDH in differential diagnoses for patients with severe neurological deficits, especially when imaging suggests neoplasms. Surgical intervention led to favorable outcomes, highlighting the importance of IDDH awareness and the need for further research into its pathogenesis and treatment guidelines.

## Introduction

Intradural disc herniation (IDDH) is a rare condition, comprising less than 0.5% of herniated disc cases, primarily in the lumbar region [1]. The first documented case was reported by Walter Dandy in 1942, who observed a herniated lumbar disc penetrating the dura mater [2]. This finding was initially suspected to be an intradural tumor, which shifted the diagnostic perspective for intradural pathologies.

The exact mechanism of IDDH remains unclear. Theories suggest that chronic aseptic erosive inflammation, congenital anomalies, minor trauma, or previous surgeries may contribute to the penetration of the dura by herniated disc material [1]. Although many theories indicate the chronic nature of the pathology involved, patients with IDDH typically present with severe nerve compression or even cauda equina syndrome [3].

There is no specific clinical examination or symptom that can predict the presence of IDDH. The gold standard for diagnosing a herniated disc is MRI. Several signs, such as the "hawk-beak" sign on axial T2 scans, the “Y-sign,” and the “Crumble Disc Sign,” have been suggested as indicative of IDDH but cannot diagnose it accurately [4-6]. MRI may reveal a severe form of herniated disc that could be mistaken for an intradural tumor [1]. Intraoperative findings may provide clues about the pathology's nature; however, a definitive diagnosis is typically obtained postoperatively through histopathological examination.

Treatment for IDDH generally involves surgical intervention due to the severe compression of neural structures. The surgical approach typically includes laminectomy and durotomy to access and remove the herniated disc material. Postoperative outcomes are generally favorable, with significant improvements in pain and neurological function reported in most cases. Conservative treatments are usually insufficient due to the intradural location of the herniation and the severity of symptoms [7,8].

## Case presentation

History and presentation

A 60-year-old female with a history of hypertension, dyslipidemia, stroke, and hypothyroidism presented with severe lower back pain, progressive lower limb weakness, and urinary retention. On examination, she exhibited bilateral lower limb weakness with hyperreflexia predominantly on the right side.

Imaging

**Figure 1:**
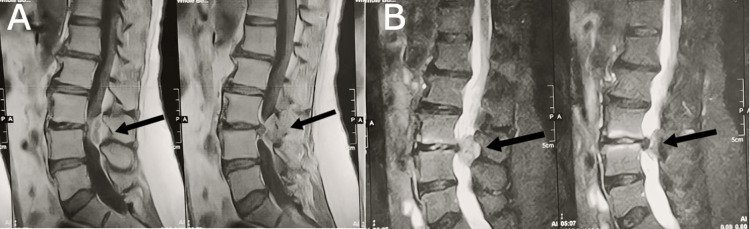
(A) Contrast-enhanced T1 MRI images of the lumbar spine show L3-4 disc prolapse associated with retrolisthesis. There is a dorsal, dural-based mass with an enhancing capsule. The core of the mass is non-enhancing. (B) T2-weighted images show the mass to be isointense. The patient presented with acute symptoms of cauda equina compression exacerbation. Based on this presentation, the decision was made to proceed with surgical excision of the mass. It was believed that the patient had two distinct pathologies: spinal instability and discogenic compression, which acutely worsened the chronic neoplastic compression. Although the mass is separated from the prolapsed disc by the enhancing capsule, the disc is in contact with the mass at the lateral area.

MRI demonstrated a posterior dural-based mass causing severe cauda equina compression, accompanied by lumbar instability at the L3-L4 level (Figure 1).

CT angiography (CTA) on neck vasculature revealed bilateral carotid artery stenosis, and echocardiography showed mild non-significant left ventricular hypertrophy.

Surgical intervention

**Figure 2:**
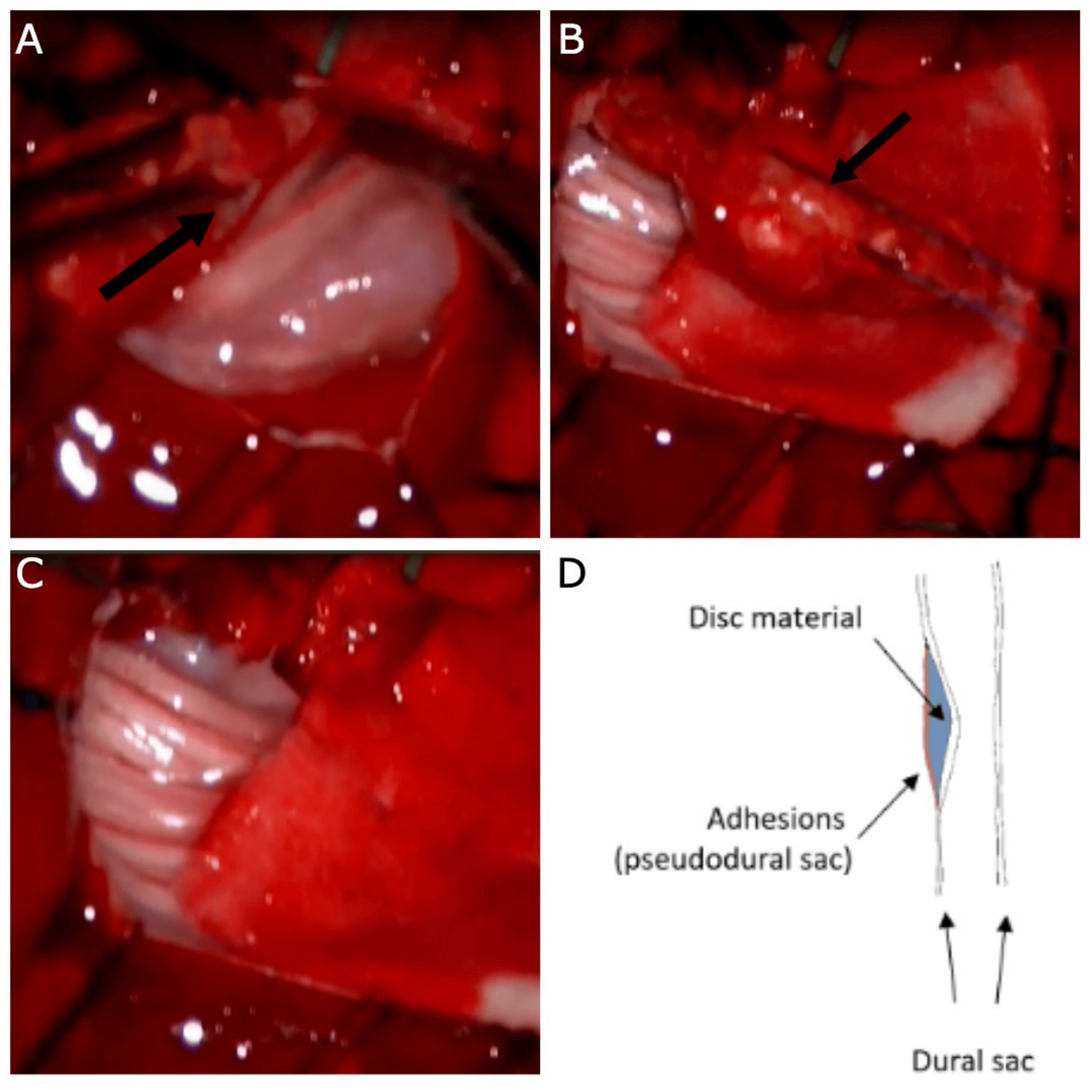
Steps involved in the removal of the dural mass (sequestrated disc material) and a diagrammatic illustration of the findings. (A) Dissection of the caudal end of the mass by incising the dorsal dura. (B) Exploration of the cranial end of the mass. (C) Complete excision of the mass along with the attached dorsal dura. (D) Diagrammatic representation of the pathology of the intradural disc, showing the disc material enclosed between the dural sac and adhesions, which form a false continuation of the dura.

The surgical procedure involved a lumbar incision, muscle dissection, spinal fixation, laminectomy, and dural opening. Intraoperatively, an intradural disc was suspected due to adhesions between the disc and dura surrounding the nerve root and cauda equina. However, a dural-based tumor could not be definitively excluded at that time. Consequently, excision of the dura along with the dural-based mass was performed (Figure 2), followed by duroplasty using a dorsal lumbar fascia graft (Video 1 of Appendices). Closure was achieved in layers, with the placement of a subfascial closed suction drain, which was removed on the second postoperative day.

Histopathology

**Figure 3:**
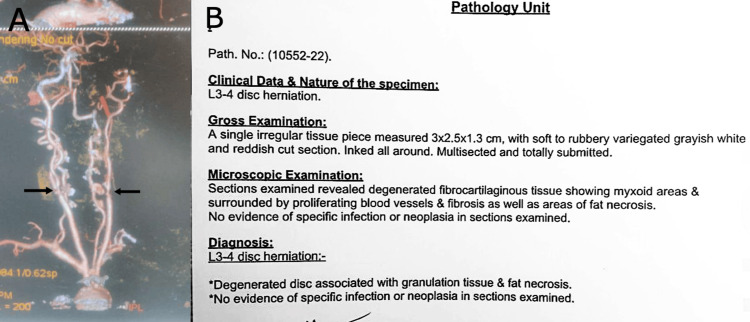
(A) CTA of the carotid arteries revealed bilateral carotid artery stenosis. (B) The pathology report of the resected mass from the L3-4 disc herniation specimen showed degenerated fibrocartilaginous tissue with fibrosis, fat necrosis, and granulation tissue, without evidence of infection or neoplasia. CTA, CT angiography

Histopathological examination confirmed the presence of disc material. A postoperative review of findings suggests that the dura had been invaginated by the sequestrated disc material, while the outer portion of the disc was encapsulated by a false dura formed from chronic adhesions (Figure 3).

Postoperative outcome

Post-surgery, the patient experienced significant pain relief, regained walking ability, and discontinued wheelchair use. Motor function and pain levels improved to pre-symptomatic levels.

## Discussion

IDDH is a rare condition where the disc herniates into the dura mater. IDDH is rare, with less than 0.5% of cases involving the lumbar spine. It is most common at L4/5, followed by L3/4 and L5/S1, and occurs predominantly in older males [9-11].

Theories of the pathogenesis of intradural discs include dural defects, chronic inflammation, and ischemic compression. These mechanisms may act synergistically, with chronic untreated and sequestrated disc herniation contributing to IDDH [1].

In our case, the latter theory predominated, as the patient reported untreated disc herniation over time, without prior surgery or significant congenital anomalies or trauma history. Importantly, a clear fibrovascular tract connecting the disc to the dura was observed during surgery.

Compression of the nerve root by the disc leads to ischemic radiculopathy, triggering neovascularization and the formation of fibrovascular tissue aimed at healing the annular tear, contributing to dural adhesions.

The herniated disc may become enveloped by fibrovascular tissue originating from the dura and adjacent vascular structures, forming a pseudo-capsule that mimics intradural herniation (Figure 2). This means that the intradural disc is not really intradural. It instead invaginated into the dura and covered on the outer layer by a pseudocapsule formed from adhesions, which developed as a result of chronic inflammation caused by prolonged compression and ischemia. The enhancing capsule, seen in Figure 1, represents the fibrovascular tissue as a hallmark of chronic inflammation.

Recent literature suggests that understanding chronic inflammatory processes and anatomical predispositions, such as congenital adhesions or prior surgeries, can aid in the early identification and management of IDDH. The role of advanced imaging techniques and intraoperative findings is crucial in differentiating IDDH from other intradural pathologies, thereby guiding appropriate surgical interventions [12].

## Conclusions

Lumbar IDDH is a rare and complex condition. The case underscores the importance of considering IDDH in patients with severe neurological deficits and chronic disc disease. Timely surgical intervention is recommended for suitable cases of disc disease. Further research is needed to understand the pathophysiological mechanisms and establish evidence-based treatment guidelines.
